# Spatially Enabling the Health Sector

**DOI:** 10.3389/fpubh.2016.00243

**Published:** 2016-11-04

**Authors:** Tarun Stephen Weeramanthri, Peter Woodgate

**Affiliations:** ^1^Department of Health, Government of Western Australia, Perth, WA, Australia; ^2^Cooperative Research Centre for Spatial Information, Carlton, VIC, Australia; ^3^Global Spatial Network Board, Cooperative Research Centre for Spatial Information, Carlton, VIC, Australia

**Keywords:** spatial information, health sector, health reform, health information, innovation, technology, end-user development

## Abstract

Spatial information describes the physical location of either people or objects, and the measured relationships between them. In this article, we offer the view that greater utilization of spatial information and its related technology, as part of a broader redesign of the architecture of health information at local and national levels, could assist and speed up the process of health reform, which is taking place across the globe in richer and poorer countries alike. In making this point, we describe the impetus for health sector reform, recent developments in spatial information and analytics, and current Australasian spatial health research. We highlight examples of uptake of spatial information by the health sector, as well as missed opportunities. Our recommendations to spatially enable the health sector are applicable to high- and low-resource settings.

## The Impetus for Health Sector Reform

Spatial information describes the physical location of either people or objects, and the measured relationships between them. We argue in this article that well-established geographic information systems (GIS), as well as more recent advances in spatial technologies, analytics, and visualization, have the potential to enrich our understanding of health systems and drive strategies for health sector reform.

In defining “health systems,” the World Health Organization (WHO) includes “all the activities whose primary purpose is to promote, restore and/or maintain health” as well as associated “people, institutions and resources.”^1^ The use of the word “sector” emphasizes the industry aspects, including its organization (public and private), financing, and performance dimensions. Health sector reforms have been defined as “sustained, purposeful changes to improve the efficiency, equity and effectiveness of the health sector” (1).

There are a number of pressures on health systems that are present in many countries, regardless of their level of development. These include an aging population, with an increasing burden of chronic disease; introduction of new technologies (services, drugs, and medical devices); and increased consumer expectations (which tend to rise with increasing wealth). In short, demand for health services is increasingly outstripping supply, and as a result, costs are rising in an unsustainable way. In the five decades to 2008, spending on health care grew by 2% points in excess of GDP growth across all Organisation for Economic Cooperation and Development (OECD) countries. From 2005 to 2009, average annual health spending growth across the OECD was 3.4%, though this slowed to 0.6% in the period from 2009 to 2013, in the aftermath of the global financial crisis (2).

Senkubuge and colleagues (3) argue that there is no single global or regional policy formula for health sector reform and that it will depend on a country’s history, values, and culture. On the one hand, many developed countries are approaching health reform from a cost-efficiency perspective, aiming to reduce costs while maintaining quality of care and improvements in longevity. On the other hand, there is a strong agenda for change in less wealthy countries, which emphasizes universal health insurance, equity, strengthening of primary care, and addressing of social and environmental determinants of health.

The WHO describes how a well-functioning health information system allows for reliable and timely decision making at different levels of the health system (4). It outlines three domains of health information: health status; determinants of health; and health system performance. These three domains have been used by many countries, including Australia, to develop performance frameworks,^2^ which can also then serve as ways to measure the success or otherwise of health reform efforts.

Having made the argument that information is a key pillar for health reform, we now turn to the value of spatial information in particular.

## The Future is Spatial, but Health Sector Uptake is Patchy

Spatial information is a broad term that describes the connection between data on positioning and location with that of people, objects (both built and natural), and activities. It includes tools such as aerial and satellite remote sensors, Global Navigation Satellite Systems (e.g., GPS), and computerized GIS software. Traditionally spatial data have been uniquely characterized as geographic (e.g., longitude and latitude) or map-based coordinates. In more recent years, the concepts of location and place (“the railway station” and “my home,” respectively) have gained broader attention. For example, “what3words”^3^ has divided the world into a 3 m-by-3 m grid and assigned a unique combination of three words to each grid cell to enable a fine granularity of positioning using language, not coordinates. So “spatial” is not “just maps.”

Spatial analytics is also changing. Traditional approaches using GIS software have typically undertaken distance and proximity analyses on single and multiple datasets that are co-registered and “stacked” in a single database. More recently, there has been a trend for spatial analytics to become increasingly web-based, accessing both data and tools from multiple sources.

Adoption of spatial technology by the public *via* Internet-enabled and mobile devices is now commonplace, and health professionals are no exception in terms of their use for personal purposes. Case studies of potential uses in the health sector include mapping and matching of needs and services, and evaluation of outcomes (including adverse events and medical errors), depending on location of residence or work (5). For example, a recent study looked at the variation in common hospital procedures (such as arthroscopies, cesarean sections, and cardiac procedures) across regions in Australia and found that much of the variation was unwarranted, unrelated to demonstrable need, and hence not a good use of scarce resources (6).

Spatial tools have also been used for many years to explore environmental determinants of cancer, describe risk factors for chronic disease, investigate disease transmission, and plan for and respond to natural disasters, including in low-resource settings, where application to infectious disease surveillance and outbreak response predominates (7). Indeed, modern public health, in the English-speaking world, was founded in the work of John Snow and his carefully drawn cholera maps in the London of the 1850s (8).

Our argument is that despite all of the above, there is little evidence that such technologies are being used in a comprehensive or planned way across the health sector to drive health reform. Certainly, in developed economies, the hospital and acute care sector has seen the benefits of precision location technology through sophisticated *within the body* imaging techniques (CT, MRI, etc.). But this has not translated into a consistent desire for location precision *outside the body*.

As a result, good spatial health practice remains the exception rather than the rule, GIS practitioners remain relatively isolated from other information analysts, the role of location or spatial information is rarely discussed at an executive level in health organizations, and it is often neglected in strategic planning. We miss many opportunities to utilize spatial technology in the health sector, and its potential remains significantly under-utilized.

## Australasian Spatial Health Research

The geoservices market is dynamic and expanding faster than the global economy (9). Many sectors, including defense, engineering, transport, energy, agriculture, environment, and mining and resources, have been utilizing spatial technology in a systematized way for many decades, and several were instrumental in a successful bid in 2003 for a Cooperative Research Centre in Spatial Information (CRC-SI).^4^ The Australian Government’s Cooperative Research Centre’s Programme^5^ supports industry-led collaborations between researchers, industry, and the community, and forms part of the National Innovation and Science Agenda.^6^ In 2010, a Health Program was included in a successful second-phase funding bid of the CRC-SI.

The CRC-SI aims for end-user-driven research, and this same principle underpins the Health Program, which has major programs on visualization and privacy protection (based in Western Australia), spatial statistical modeling (based in Queensland), and healthy cities and recovery from natural disasters (based in New Zealand).^7^

It is helpful for end-users if spatial tools are available and accessible to more than a few GIS specialists. Therefore, the Department of Health in Western Australia (DoHWA) has developed an online geovisualisation tool called *HealthTracks*, with both interactive mapping and reporting functions, that makes a broad range of demographic, health, and environmental data available *via* a web interface to all employees (10). Non-expert users, including clinicians, program managers, policy makers, and planners, can generate their own local area reports, tables, and maps.

*HealthTracks* has successfully extended the number of users of GIS information in DoHWA. Prior to its development, around six epidemiologists and GIS analysts were regular users of spatial technologies. The semi-automation of analytics, coupled with a largely plain language interface, has seen 150 users generate over 7000 maps and reports in the last year. However, this number remains a fraction of the total workforce of over 40,000.

A related project called *Epiphanee* has focused on generating dynamic privacy protections, based on a complex algorithm that takes into account small number analysis and reporting – a particularly important issue for health data, and of particular concern to data custodians charged with protecting health privacy (11). The program allows the user, when making a single data request from multiple datasets, to trade-off competing dimensions of area size, disease specificity, and demographic composition, and generate a report that falls within probabilistic privacy limits set by the data custodians. This project highlights the distinctive and highly sensitive nature of health information.

A good spatial analytics program for health relies on fundamental spatial concepts, including scale, accuracy, and geocoding uncertainty (12), as well as a critical approach to spatial thinking, reasoning, and language (13). Data analysis also requires specialized statistical expertise to deal with the heterogeneity of spatial data, and its tendency to exhibit autocorrelation. Using the release of the Atlas of Cancer in Queensland (14) as a foundation, Queensland researchers have developed new spatiotemporal modeling techniques to examine cancer incidence and survival within small areas, important for understanding and reducing population-level inequities. Analysis based on the Atlas led the Queensland government to double its rural travel subsidy for patients to attend screening and treatment facilities.

Epidemiological studies have traditionally looked at the aggregate relationship between geographic areas and disease risk factors or outcomes (e.g., a certain neighborhood may have an elevated level of obesity or diabetes). Longitudinal spatial studies are much less frequent. In New Zealand, University of Canterbury researchers have used fine-grained spatial tools and modeling, to examine the medium to long-term health and mental health impacts of the 2010/2011 earthquakes, particularly as they relate to place of exposure and subsequent mobility (15). The New Zealand research builds on a joint Ministry of Health-University of Canterbury venture, called the GeoHealth Laboratory (16), a partnership that helps ensure research results inform the targeting and ongoing design of social support and health services.

Even less frequently explored is how geocoded social determinants can be used to improve patient care at the community health center level (17). Research has identified the spatial clustering of older patients with poorly controlled diabetes within a large Australian general practice using individual-level data (18). Such analyses have the potential to promote new preventive strategies and stimulate better targeted approaches to patient management at a community level.

Australia has also focused on building capacity through international partnerships and is a member of the Global Spatial Network (GSN),^8^ which has identified the health sector as a priority sector for growth and application of spatial technology. The GSN is made up of research organizations that specialize in collaborative research. Member organizations must have partners drawn from the research sector, the private sector, and the government sector. Partnering organizations have come from Sweden, the EU, the US, Canada, Mexico, Korea, New Zealand, and Australia. The GSN seeks to promote international collaboration in complex, multi-organization spatial research and facilitate information sharing. As a result of the Network’s activities, Sweden now hosts an annual workshop of spatial and health researchers as part of their “Geolife Region program.”^9^

Training is central to building capacity, and the teaching of spatial skills encompasses much more than the use of GIS software. Specially commissioned special “GIS Awareness for Health Professionals” training courses that use case studies and scenarios to promote the intelligent application of spatial data and analysis within the health sector have now begun to be developed.^10^

These projects and products have not yet, however, been game changers in the health sector in Australia. There are a number of factors, including the relatively modest size of the program (less than one million Australian dollars per year), and its positioning as a “research” rather than a “services” program within a very large industry. But there may be other factors. In thinking through this issue, we adopted a model of technology use as the product of “context, tool and user,” and a “spatial maturity” model as a *set of capabilities* required for the effective use of spatial technology (19).

The CRC-SI has developed and trialed this model in a health sector setting, in order to benchmark performance and drive organizational improvement. The messages were clear: there is more to technology adoption than just “cool tools”; use of a generic framework can help assess and improve “capacity to use”; and mixed methods analysis (combining quantitative and qualitative approaches) can generate critical organizational insights (20).

## Challenges and Future Directions

Spatial technology encompasses much more than static maps. The global public are already familiar with an array of new dynamic location tools from GPS-enabled mobile phones to Google Earth. Newer trends in data sharing made possible through wearable sensors, crowdsourcing, and interactive social media platforms are developing quickly and will stimulate debate about the social and contextual dimensions of *place*, to complement the technical considerations of *space* and *location* (21).

Developments in remote sensing and positioning technology will provide more precise environmental data, wearable sensors will provide a wealth of individual data, and informatics will allow the analysis of such big and complex datasets in much closer to real time. Importantly, computing power has increased, and technology costs have fallen, leading to potential applications even in low-resource settings.

There is likely to be increasing public demand for more tailored risk communication and personalized medicine, which will depend, in part, on more accurate and accessible spatial data. But there is no guarantee that advances in the use of spatial technology for individuals will aggregate neatly into improvements at a population level. In other words, precision medicine or precision “wellness” that is available only to a minority, and does not give consideration to other determinants of health, can aggravate inequity in a population (22). Hence, there is need for a broader performance framework for successful health reform.

In the health sector, uptake of spatial technology will also be affected by parallel developments in e-Health, telehealth, business intelligence, “Big Data,” and the web (3.0 and beyond). Over time, more datasets will be linked, more health information will be available online, and more use made of off-the-shelf software and automated processing. As governments make more of their data open and freely available, the potential to combine such data with open analytic tools and personal data from other sources will also increase, leading to potentially greater insights but also foreseeable dangers. Privacy, data sharing, and data security concerns will need to be continuously addressed. In this new environment, the semantic web^11^ has the potential to empower users to access sophisticated programs through plain language queries. For example, it may be feasible to perform a geographic search for all cancer screening facilities within a radius of a chosen location and combine this with patient screening behavior, sociodemographic information, and cancer outcomes, so as to better target interventions to increase screening rates – all from a web browser.

## Missed Opportunities

The public, clinicians, health system planners, and policy makers each have a stake in improving both the spatial specificity of the information that underpins advanced analytics and our ability to visually communicate that information for a variety of purposes, including risk communication, service delivery, and planning, and policy.

However, we think that much more needs to be done to catalyze a transformation of what is a massive and complex industry sector, so that spatial information can become integral to evidence-based, data-rich, and patient-centered health reform. Supporting spatial data infrastructures (such as the European Union INSPIRE^12^ initiative) are well established in some regions, but it is the culture and capabilities within the health sector that are poorly developed.

As a result, the health community misses clear opportunities to add value to information from a spatial perspective. Two recent Australian examples are the Personally Controlled Electronic Health Record^13^ and the National Disability Insurance Scheme.^14^ Neither of these large potentially transformative national programs, critical to health sector reform, considered or built detailed spatial specifications into their initial roll out plans.

We would like to see such initiatives, and indeed all large agencies that handle health data, undertake a “spatial maturity” review to identify their existing operational capabilities, and any measures that could be readily adopted or adapted to improve information handling and analysis in a systematic way and in a strategic context. This would transform the potential of spatial information – from an optional extra to an essential ingredient of a strong information strategy underpinning health reform. Spatial maturity reviews also serve as a form of future due diligence, setting up a pathway that links agencies to both the activities of the spatial analytics research community and the proprietary tools of the private sector.

## Recommendations

In summary, based on our experience in health delivery and spatial health research, we believe that the core technology is present and developing rapidly for spatial information to contribute to health sector reform. No major technological breakthrough is needed. What is missing is an attitude change to see the potential and make the most of spatial data and analytics, as well as to incorporate spatial thinking into strategic thinking.

We therefore make the following recommendations to spatially enable the health sector:

(a) continue to communicate strong case studies of where spatial technology has led to improved decision making and value for end-users, mapped against key health performance areas;
(b) formally evaluate the use, costs, and benefits that the technology provides in the health sector and test its applicability in high- and low-resource settings;
(c) use spatial data to describe patient pathways, particularly for common chronic conditions, across an integrated health system from community to primary care to hospital and back;
(d) explicitly consider the “spatial maturity” model as a tool to drive organizational change;
(e) commission national-level studies that focus on pathways to adoption of spatial technology that includes major health industry stakeholders;
(f) learn the lessons from other major industry sectors, which are more advanced in their use of spatial information;
(g) include spatial information and supporting technologies when developing broader information, digital health, and “big data” strategies; and
(h) build training, capacity, and new stakeholder partnerships, nationally and internationally.

## Author Contributions

TW created the first draft of this article, based on a set of discussions with PW over many years. Both TW and PW have contributed ideas, text, and references to subsequent and final versions.

## Conflict of Interest Statement

The authors declare that the research was conducted in the absence of any commercial or financial relationships that could be construed as a potential conflict of interest. The reviewer MP and handling Editor declared their shared affiliation, and the handling Editor states that the process nevertheless met the standards of a fair and objective review.

## Footnotes

^1^http://www.who.int/healthsystems/hss_glossary/en/.

^2^http://meteor.aihw.gov.au/content/index.phtml/itemId/435314.

^3^http://what3words.com/.

^4^http://www.crcsi.com.au/.

^5^http://www.industry.gov.au/industry/IndustryInitiatives/IndustryResearchCollaboration/CRC/Pages/default.aspx.

^6^http://www.innovation.gov.au/page/agenda.

^7^http://www.crcsi.com.au/research/4-4-health/.

^8^http://www.globalspatial.org/.

^9^http://geoliferegion.com/about-geo-life-region/about-geo-life-region-2/.

^10^http://ngis.com.au/gis-awareness-for-health-professionals/.

^11^https://www.w3.org/standards/semanticweb/.

^12^http://inspire.ec.europa.eu/.

^13^http://www.health.gov.au/internet/main/publishing.nsf/content/ehealth-record.

^14^https://myplace.ndis.gov.au/ndisstorefront/index.html.
